# Bundle for gastric catheterization in newborns: from construction to validation

**DOI:** 10.1590/0034-7167-2024-0360

**Published:** 2025-06-20

**Authors:** Rubinéia Stefania da Silva, Tainá Vilhar Siqueira, Maria Paula Custodio da Silva, Karoline Faria de Oliveira, Elizabeth Barichello, Mariana Torreglosa Ruiz, Divanice Contim

**Affiliations:** I Universidade Federal do Triângulo Mineiro. Uberaba, Minas Gerais, Brazil

**Keywords:** Newborn, Gastrointestinal Intubation, Neonatal Intensive Care, Nursing Care, Feeding Methods., Recién Nacido, Intubación Gastrointestinal, Cuidados Intensivos Neonatales, Cuidados de Enfermería, Métodos de Alimentación.

## Abstract

**Objective:**

To describe the process of constructing and validating a bundle for gastric catheterization in newborns.

**Methods:**

Methodological study, developed between April and October 2023, in three stages: literature review, construction and content validation by 23 experts. The bundle for validation, composed of six items presented in Likert format, was evaluated online by the experts. The Content Validity Index with values above 0.80 were considered for analysis.

**Results:**

The final version of the bundle consisted of four items. The proposed care is related to measuring the length for insertion of the gastric catheter and methods for verifying its adequate positioning in newborns. The overall assessment of the bundle presented an overall score of 10.0.

**Conclusion:**

The validation and implementation of the bundle may contribute to the quality of care, aiming at evidence-based practice and reduction of adverse events, classifying its importance and applicability for the procedure.

## RESUMEN

**Objetivo:**

Describir el proceso de construcción y validación de un paquete para cateterismo gástrico en recién nacidos.

**Métodos:**

Estudio metodológico, desarrollado entre abril y octubre de 2023, en tres etapas: revisión de literatura, construcción y validación de contenido por 23 expertos. El paquete de validación, que consta de seis ítems presentados en formato Likert, fue evaluado en línea por expertos. Se consideró para el análisis el Índice de Validez de Contenido con valores superiores a 0,80.

**Resultados:**

La versión final del paquete constaba de cuatro elementos. Los cuidados propuestos están relacionados con la medición de la longitud para la inserción del catéter gástrico y métodos para verificar su adecuado posicionamiento en los recién nacidos. La evaluación global del paquete presentó una puntuación global de 10,0.

**Conclusión:**

La validación e implementación del paquete puede contribuir para la calidad de la atención, apuntando a una práctica basada en evidencia y reduciendo los eventos adversos, clasificando su importancia y aplicabilidad para el procedimiento.

## INTRODUCTION

Neonatology is characterized by its specificities and peculiarities, which require specialized care. Over the past decades, technical and scientific advancements, along with improvements in work processes, have significantly contributed to increased survival rates of newborns (NBs) admitted to neonatal intensive care units (NICUs) ^(1,2)^.

In NICUs, prolonged hospitalizations are common, during which neonates are frequently subjected to various interventions performed by different professionals, in addition to excessive handling and invasive procedures. This scenario can compromise patient safety, making neonates more susceptible to adverse events ^(3)^.

Gastric catheterization is a common procedure performed in hospital settings, particularly in NICUs. This procedure, exclusively performed by nurses, is not without risks and involves a series of decisions that may compromise neonatal safety during hospitalization ^(4,5)^.

Gastric catheters (GCs) are used for several purposes, including gastric decompression, medication administration, gastric lavage, gastric rest after surgeries, bleeding monitoring, and feeding. Their primary indications for neonates focus on NBs with immaturity and/or a lack of coordination in sucking, swallowing, and breathing, as well as those presenting with tachypnea or dyspnea. These recommendations aim to minimize the risk of aspiration, ensuring a safe and effective approach to the use of GCs in neonates ^(6)^.

Incorrect positioning of the GC is the leading cause of complications associated with the catheterization procedure. Studies in neonates and children show a significant prevalence of adverse events, ranging from 47.5% to 59%. Improper positioning may result in respiratory complications and nutritional deficits during hospitalization. Additionally, trajectory errors during catheter insertion, including deviation into the tracheobronchial tree and tissue perforation, represent a critical category of adverse events with the potential to cause severe and, in some cases, fatal complications for neonates ^(3,7)^.

The development and implementation of standardized institutional protocols, based on the latest patient-centered scientific evidence, play a crucial role in achieving appropriate and harm-free care. These protocols enable the effective management of processes, tasks, and activities ^(8)^.

Ensuring safe care for neonates in NICUs requires evidence-based protocols and strategies that optimize processes and outcomes. In this context, care bundles-standardized interventions based on best practices-are essential for promoting safety and efficacy in care delivery. When applied in a coordinated manner, these bundles reduce adverse events and improve clinical outcomes, particularly in preventing incidents related to the correct positioning of GC ^(9,10)^.

## OBJECTIVE

To describe the process of developing and validating a bundle for gastric catheterization in NB.

## METHODS

### Ethical Aspects

To comply with Resolution 466/2012 of the National Health Council, which regulates research involving human subjects, all participants signed an Informed Consent Form (ICF). The study was approved by the Research Ethics Committee, in accordance with the criteria outlined in Resolution 466/2012.

### Type and Period of the Study

This methodological study was conducted in three stages: a review of scientific literature on the topic, the development of a bundle for GC care in NB-including measurement and verification of the GC-and content validation performed by experts ^(11)^. The study followed the Standards for Quality Improvement Reporting Excellence ^(12)^. It was conducted from October 2022 to January 2024 at a public university in Minas Gerais, Brazil.

### Population, Inclusion, and Exclusion Criteria

Experts were recruited using the Lattes Platform, identifying 254 nurses with experience in neonatology and/or pediatrics. After analyzing curricula based on predetermined criteria, 28 nurses met the eligibility requirements.

To form the evaluator group, the curricula were assessed using specific scoring criteria: four points for a minimum of four years of clinical experience in the study area (mandatory); one point for at least one year of clinical teaching experience in the study area; one point for published articles; one point for at least two years of participation in research groups in the study area; two points for a doctoral degree in the study area; one point for a master’s degree in the study area; and one point for residency in the study area. Additional points were awarded for each additional year of clinical or teaching experience. Experts were categorized as junior specialists (minimum of five points), master specialists (six to 20 points), or senior specialists (more than 20 points) ^(13)^.

Experts were contacted via email and provided with a document detailing the study objectives and requested activities, along with the ICF and a link to the Google Form. After agreeing to participate, they were given access to the instrument. Refusals led to the termination of the process. Three experts were excluded for failing to return the instrument within 15 days, and two were excluded for incomplete responses. Out of the 28 contacted, 23 participated, forming a non-probabilistic convenience sample ^(14)^. Validation was conducted in a single round.

### Study Protocol

A theoretical-methodological framework comprising three procedural stages-theoretical, empirical, and analytical-was adopted ^(11)^. The theoretical stage involved a review of scientific literature. The empirical stage focused on developing the bundle for validation by experts, while the analytical stage involved evaluating validation results.

The instrument’s items were developed through a comprehensive search across databases including Cochrane, Latin American and Caribbean Health Sciences Literature (LILACS) via the Virtual Health Library (VHL), Medical Literature Analysis and Retrieval System Online (MEDLINE) via PubMed, Cumulative Index to Nursing and Allied Health Literature (CINAHL), Excerpta Medica database (EMBASE), and Scopus. Primary studies published in Portuguese, English, or Spanish between January 2013 and June 2023 were included. Excluded were review articles, reflections, commentaries, conference abstracts, theses, dissertations, opinion articles, undergraduate projects, editorials, reports, official program documents, book chapters, and e-books.

The review question was: “What evidence exists in the literature regarding GC measurement techniques, and what alternative methods to radiological examination are available for verifying correct catheter placement in NB admitted to NICUs ?”

Searches were performed using controlled Medical Subject Headings (MeSH) terms: “Infant, Newborn,” “Intubation, Gastrointestinal,” and “Intensive Care, Neonatal,” as well as Health Sciences Descriptors (DeCS): “*Recém-nascido*,” “*Intubação gastrointestinal*,” and “*Terapia Intensiva Neonatal*,” combined with Boolean operators “OR” and “AND.” These terms were validated by a librarian. Searches were conducted between April and June 2023, with results independently reviewed by two researchers. In cases of disagreement, a third researcher provided input, and decisions were made by consensus.

Based on the evidence, the validation instrument was developed using HyperText Markup Language (HTML) in Google Forms®, with a 30-day response deadline. The instrument consisted of two sections. The first part addressed information for characterizing and classifying the specialists, according to the following criteria ^(13)^: age, gender, length of professional experience, clinical experience, length of nursing teaching, improvements and specializations in the neonatal field, and publications in the area.

The second part constituted the criteria for analyzing the items that made up the bundle, based on specific statements ^(11)^, including: (1) the instrument is applicable and has clear and feasible instructions; (2) the recommendations allow achieving the desired objective; (3) the items express a single idea and allow adequate understanding; (4) the content is explained clearly and unequivocally; (5) the instrument is relevant and meets the proposed purpose; (6) each item of the instrument is distinct from the others and is not confused; (7) the language is appropriate and allows interactivity of the content; (8) the vocabulary is appropriate, without generating ambiguities; (9) the vocabulary is consistent with the theme, with appropriate concepts; (10) the formulation of the instrument contributes to a favorable attitude towards using and understanding the content; (11) The content is current, consistent, and sufficiently in-depth for understanding the topic; (12) The proposed sequence is balanced and coherent. The evaluation was conducted using a Likert scale with the following options: strongly agree (4), agree (3), neutral (0), disagree (2), and strongly disagree (1). At the end of each item, a blank field was provided for experts to comment on the bundle’s practical utility and offer “comments or suggestions” regarding semantic alignment for removal, additions, and/or modifications to each item. ^(11)^

### Analysis of Results and Statistics

The data were exported from Google Forms® to an Excel® database, undergoing a double-entry process by two independent researchers to ensure data reliability. The Content Validity Index (CVI) was used to evaluate expert consensus regarding the representativeness of care items to be included in the instrument. The CVI was calculated by summing responses of “strongly agree” or “agree” and dividing by the total number of responses. The consensus threshold among experts was set at 80%, as recommended by the literature ^(14)^.

## RESULTS

The study included 23 experts, all specializing in neonatology and/or pediatrics (100%). The majority were female (91.3%), with an average age of 38 years (minimum 29, maximum 57 years). The mean professional experience was 12 years. Based on professional experience, 21 experts (91.3%) were classified as master specialists, and two (8.7%) as senior specialists.

Regarding academic qualifications (participants could select more than one option), two held undergraduate degrees (8.7%), 17 had specializations (74%), six had residency training (26%), five held a master’s degree (21.7%), four had a doctoral degree (17.4%), and one had a postdoctoral degree (4.3%).

The initial instrument developed for validation comprised six items on gastric catheterization in NBs, aimed at reducing issues related to measurement and verification of proper catheter placement, as shown in chart 1.

**Chart 1:** Components of the Bundle for Reducing Issues Related to gastric catheter Measurement and Positioning in NB (Validation Version). Uberaba, Minas Gerais, Brazil, 2023

Components
Verify whether the newborn requires GC insertion
Perform hand hygiene before and after the procedure.
Use the NEMU technique for catheter measurement: If oral insertion: measure from the lip commissure to the lower lobe of the ear and to the midpoint between the xiphoid process and the umbilical scar. If nasal insertion: measure from the tip of the nose to the earlobe and to the midpoint between the xiphoid process and the umbilical scar.
Verify catheter positioning by aspirating gastric contents to examine the color and perform a pH test.
Perform an X-ray to confirm catheter placement, if necessary
Document the procedure and occurrences in the nursing record.

*GC: gastric catheter*

Inter-evaluator agreement was demonstrated for the items included in the proposed bundle and for the 12 content evaluation criteria ^(11)^, as presented in Table 1.

**Table 1:** Distribution of Expert Responses, Content Validity Index by Criterion, and Overall Content Validity Index for the Bundle to Reduce Issues Related to gastric catheter Measurement and Positioning in NB. Uberaba, Minas Gerais, Brazil, 2023

Bundle itens General Evaluation Criteria	Likert Scale Response Options					Responses 3 and 4	CVI^*^
	0 n(%)	1 n(%)	2 n(%)	3 n(%)	4 n(%)	n(%)	(%)
Clear and actionable			1(4.3)	10(43.5)	12(52.2)	22(95.7)	0.96
Achieves the objective				10(43.5)	13(56.5)	23(100)	1.00
Comprehensible	1(4.3)		1(4.3)	9(39.1)	12(52.2)	21(91.3)	0.91
Content clarity	1(4.3)			6(26.1)	16(69.5)	22(95.6)	0.96
Relevant	1(4.3)	1(4.3)		7(30.4)	14(60.9)	21(91.3)	0.91
Items are distinct				10(43.5)	13(56.5)	23(100)	1.00
Clear for target audience		1(4.3)	1(4.3)	8(34.8)	13(56.5)	21(91.3)	0.91
Clear language for target audience				8(34.8)	15(65.2)	23(100)	1.00
Language appropriate to content			2(8.7)	8(34.8)	13(56.5)	21(91.3)	0.91
Contributes to understanding	1(4.3)		1(4.3)	9(39.1)	12(52.2)	21(91.3)	0.91
Current content	1(4.3)		1(4.3)	8(34.8)	13(56.5)	21(91.3)	0.91
Coherent sequence			2(8.7)	07(30.4)	14(60.9)	21(91.3)	0.91
Total CVI†							0.94

* *CVI: Content Validity Index per item; Total CVI†: Overall Content Validity Index of the bundle*

Regarding inter-evaluator agreement, initially analyzed for each item in the proposed bundle, all evaluated criteria were classified as demonstrating “almost perfect agreement” (0.81 to 1.00). The Content Validity Index (CVI) analysis by criterion revealed that three items (25%) received the maximum score of 1.00. Two items (16.7%) achieved a score of 0.96, while seven items (58.3%) scored 0.91.

A total CVI of 0.94 (“almost perfect agreement”) was obtained for the bundle, supporting its characterization as a valid construct in terms of content. A re-evaluation by the experts was not required. Thus, the refinement of the construct, following the incorporation of evaluator suggestions, was conducted solely as a feedback process.

After reviewing the experts’ suggestions, improvements were made to the bundle to make it more practical and easier to understand. Items one and six from the initial instrument were removed because the catheterization would only be performed on neonates (NB) with clinical indications, and nursing documentation is considered mandatory for any procedure. As a result, the final version of the bundle was streamlined to four items, reflecting a more focused approach tailored to the specific needs of gastric catheterization procedures in neonates. This simplification aims to optimize the bundle’s practical application while maintaining its efficacy and relevance for the nursing team.

The final version of the bundle for gastric catheterization in neonates, aimed at reducing issues related to measurement and positioning of the GC in NICUs, is presented in chart 2, incorporating the changes suggested by the experts.

**Chart 2:** Final Version of the Bundle for Reducing Issues Related to the Measurement and Positioning of gastric catheters in Neonates. Uberaba, Minas Gerais, Brazil, 2023

Bundle for Reducing Issues Related to the Measurement and Positioning of GCs in Neonates
Perform hand hygiene before and after the procedure
Use the NEMU technique for catheter measurement, considering the total length, including the openings: For oral insertion: From the labial commissure to the lower lobe of the ear to the midpoint between the xiphoid process and the umbilical scar. For nasal insertion: From the tip of the nose to the lobe of the ear to the midpoint between the xiphoid process and the umbilical scar.
Verify catheter positioning: Aspirate gastric contents to examine color, perform a pH test (reference value: ≤5.5), and measure the exposed length of the catheter.
Perform an X-ray if there are doubts about the positioning of the GC.

*GC: gastric catheter*

## DISCUSSION

The development and implementation of bundles have proven effective in improving patient outcomes and processes while preventing health complications, particularly those considered avoidable ^(15-17)^. Adopting this approach helps standardize nursing team actions, reducing disparities in care delivery.

Validating the process adds methodological robustness to the bundle, making it crucial to evaluate the target population’s perspective to enhance understanding of its elements ^(11,18)^. Simple and straightforward tools are essential in clinical practice, considering the demands and resources available in healthcare services.

One of the key components of the bundle is “hand hygiene,” often listed as the first item in most health safety protocols. This practice is recognized as a low-cost, simple, and highly effective measure to prevent infections. However, limitations in adherence among healthcare professionals must be addressed ^(19)^.

An anatomical discrepancy exists between the distance from the tip of the nose to the earlobe for nasal catheter insertion and the distance from the labial commissure to the earlobe for oral insertion. Although this difference is minimal in neonates, it may affect proper catheter positioning. Therefore, further research is essential to validate external anatomical reference measurements for GC insertion. This approach aims to prevent complications from improper positioning and improve clinical practices related to the procedure ^(20)^.

The choice of the GC insertion route should consider each neonate’s neuromuscular coordination development. For instance, an oral gastric catheter may be used in the initial days and later replaced by a nasal GC after the baby achieves respiratory stabilization. This personalized approach ensures a safe and effective procedure tailored to each neonate’s specific needs ^(8)^.

To ensure the appropriate gastric positioning of the catheter and prevent complications, the guidelines from the Neonatal Resuscitation Program of the American Academy of Pediatrics, the National Association of Neonatal Nurses, and Brazil’s Ministry of Health, through the Good Practices Manual and Neonatal Resuscitation Manual of the Brazilian Society of Pediatrics, recommend adopting the NEMU method ^(21-23)^.

The suggested adjustments during the instrument’s validation process affected three items in the bundle, aiming to improve understanding of the care practices.

The first suggestion addressed the consideration of catheter openings. The lack of guidance on precautions for catheters with multiple distal openings in reviewed studies highlighted a significant gap. Studies mentioned “insertion length” without clarifying whether it should include or exclude openings, a critical detail that should be explicitly defined. Considering this absence, the recommendation is to measure the total length, including the openings, from the distal end. It is crucial to use a catheter specifically designed for this purpose, such as a pediatric GC, to ensure accurate measurement. Using urethral or tracheal suction catheters, for instance, may compromise accuracy due to variations in the standardization of openings ^(6)^.

The second suggestion pertained to verification methods to confirm GC positioning. Regarding accuracy and safety, alternative methods to radiological examination, such as auscultation and isolated aspiration, are not recommended. Epigastric auscultation is considered unreliable and discouraged due to the difficulty in distinguishing the sound of air instilled in the stomach from that in the lungs. Similarly, aspiration of secretions and evaluation of their color and appearance, while potentially sensitive, are imprecise due to the lack of established specificity. Endotracheal and bronchial secretions may present colors and appearances similar to gastric secretions. Combining two or more verification methods is recommended to enhance reliability ^(24,25)^.

Currently, expert consensus suggests that bedside pH testing with reagent strips is the safest and most recommended initial method for confirming GC positioning. Results indicating a pH ≤5.5 suggest correct gastric positioning, whereas values >5.5 require radiological confirmation ^(24)^.

It is worth noting that pH strips may not be readily available in some Brazilian hospitals. Therefore, selecting a method to evaluate GC trajectory and position depends on available resources and nursing care planning within each institution ^(6)^. Implementing this method in hospital clinical practice is crucial, given its recommendation and the low cost of pH strips.

The third suggestion involved using X-rays to confirm GC positioning, establishing guidelines for actions when issues are identified. In cases of uncertainty about the GC’s correct position, replacement, re-evaluation, and/or radiographic confirmation should be considered before administering any content via the catheter ^(8,24)^.

### Contribution to Nursing and Health

Literature findings reveal significant divergences in GC measurement practices and alternative methods for verifying positioning, raising concerns. These results underscore the urgency of incorporating measurement strategies and catheter position verification into institutional protocols and emphasizing the importance of continuous professional education. Such initiatives are vital to enabling changes in individual practices. This study is expected to provoke reflection and discussion about gastric catheterization practices in neonatal units, supporting nursing actions and care in neonatology.

### Study Limitation

The study’s limitation lies in the scarcity of evidence on the topic from randomized clinical trials, considered the gold standard for evaluating the efficacy and safety of health interventions.

## CONCLUSION

This study facilitated the construction and content validation of a bundle, evaluated by 23 experts, for the gastric catheterization procedure in neonates. The care practices addressed aspects related to GC insertion length measurement and correct positioning. All bundle items achieved a CVI above the recommended threshold, and minor adjustments were implemented to enhance overall comprehension. Participants recognized the instrument’s relevance to clinical practice. Adopting this bundle could contribute to improved care quality and nursing practices in neonatal care, serving as a complementary tool to the provided care.

## References

[1] André RR, Mendes KQS, Avelar AFM, Balieiro MMFG. (2017). Enteral tube placement in newborns according to the modified measurement technique. Acta Paul Enferm.

[2] Nascimento J, Santos IMM, Silva LJ. (2019). Cuidados com recém-nascidos alimentados por sonda gástrica: conceitos e práticas. Texto Contexto Enferm.

[3] Silva PNJ, Baptista SCPD, Carvalheira APP, Russo NC, Bocchi SCM. (2023). Técnicas de mensuração para sondagem gástrica em recém-nascidos: revisão integrativa. Rev Bras Rev Saúde.

[4] Dias FSB, Emidio SCD, Lopes MHBM, Shimo AKK, Beck ARM, Carmona EV. (2017). Procedures for measuring and verifying gastric tube placement in newborns: an integrative review. Rev Latino-Am. Enfermagem.

[5] Silva HR, Ferreira LF, Fernandes MTC, Dellanhese APF. (2020). Métodos alternativos de verificação do posicionamento de sonda gástrica em crianças. Saúde Colet.

[6] Souza CF, Araújo CMT, Barreto AKCP. (2022). Comprimento de inserção de sonda gástrica em recém-nascidos: práticas dos enfermeiros. Rev Enferm UERJ.

[7] Manzo BF, Marcatto JO, Ferreira B, Galvão Diniz C, Parker LA. (2023). Comparison of 3 Methods for Measuring Gastric Tube Length in Newborns: a randomized clinical trial. Adv Neonatal Care.

[8] Soares LS, Silva GRF, Machado RS. (2017). Evidências científicas sobre uso e cuidados de enfermagem com tubos orogástricos em neonatos prematuros. Rev Soc Bras Enferm Ped.

[9] Zegers M, Hesselink G, Geense W, Vincent C, Wollersheim H. (2016). Evidence-based interventions to reduce adverse events in hospitals: a systematic review of systematic reviews. BMJ Open.

[10] Reis Bellaguarda ML, Schuller Vieira IM, Petri JH, Coelho R, Kiefer Moraes CL. (2020). Bundle de prevenção das complicações da sondagem nasoenteral em unidade de terapia intensiva. Glob Acad Nurs.

[11] Pasquali L. (2010). Instrumentação psicológica: fundamentos e práticas.

[12] Ogrinc G, Davies L, Goodman D, Batalden P, Davidoff F, Stevens D. (2016). SQUIRE 2.0 (Standards for QUality Improvement Reporting Excellence):revised publication guidelines from a detailed consensus process. BMJ Qual Saf.

[13] Guimarães HCQCP, Pena SB, Lopes JL, Lopes CT, Barros ABL. (2016). Experts for Validation Studies in Nursing: New Proposal and Selection Criteria. Int J Nurs Knowl.

[14] Polit DF, Beck CT. (2019). Fundamentos de pesquisa em enfermagem: avaliação de evidências para a prática da enfermagem.

[15] Hawes JA, Lee KS. (2018). Reduction in Central Line-Associated Bloodstream Infections in a NICU: practical lessons for its achievement and sustainability. Neonatal Netw.

[16] Short KL. (2019). Implementation of a Central Line Maintenance Bundle for Dislodgement and Infection Prevention in the NICU. Adv Neonatal Care.

[17] Payne V, Hall M, Prieto J, Johnson M. (2018). Care bundles to reduce central line-associated bloodstream infections in the neonatal unit: a systematic review and meta-analysis. Arch Dis Child Fetal Neonatal.

[18] Coluci MZO, Alexandre NMC, Milani D. (2015). Construção de instrumentos de medida na área da saúde. Ciênc Saúde Colet.

[19] Cavalheiro AC, Trentino JP, Alves FC, Puggina AC. (2019). Regulatory Standard 32 ban on adornments and professional self-concept of nursing professionals. Rev Bras Med Trab.

[20] Lopes LS, Silva GD, Alves AMA, Reis AT, Silva GRG, Silvino ZR. (2019). Cateterização gástrica em recém-nascidos prematuros: análise de prevalência das técnicas de mensuração. Rev Enferm UERJ.

[21] Clifford P, Heimall L, Brittingham L, Davis KF. (2015). Following the evidence: enteral tube placement and verification in neonates and Young children. J Perinat Neonatal Nurs.

[22] Sociedade Brasileira de Pediatria (SBP) (2021). Reanimação do recém-nascido ≥34 semanas em sala de parto: diretrizes da Sociedade Brasileira de Pediatria.

[23] Ministério da Saúde (BR) (2019). Principais Questões sobre Cuidados com o Recém-nascido na UTI Neonatal.

[24] Dias FSB, Almeida BP, Alvares BR, Jales RM, Caldas JPS, Carmona EV. (2019). Use of pH reagent strips to verify gastric tube placement in newborns. Rev Latino-Am Enfermagem.

[25] Rodrigues da Silva H, Ferreira LP, Fernandes MTC, Dellanhese APF. (2020). Métodos alternativos de verificação do posicionamento de sonda gástrica em crianças. Saud Coletiv.

