# Supplementary material for: Bundle for gastric catheterization in newborns: from construction to validation
Source: Rev Bras Enferm. 2025 Jun 20;78(2):e20240360. doi: 10.1590/0034-7167-2024-0360 (PMC12185143; doi:10.1590/0034-7167-2024-0360)
Supplement: Translation

# Translation: *Bundle* para cateterização gástrica em recém-nascidos: da construção à validação

**DOI:** 10.1590/0034-7167-2024-0360pt

**Authors:** Rubinéia Stefania da Silva, Tainá Vilhar Siqueira, Maria Paula Custodio da Silva, Karoline Faria de Oliveira, Elizabeth Barichello, Mariana Torreglosa Ruiz, Divanice Contim

**Affiliations:** I Universidade Federal do Triângulo Mineiro. Uberaba, Minas Gerais, Brasil

**Keywords:** Recém-Nascido, Intubação Gastrointestinal, Terapia Intensiva Neonatal, Cuidados de Enfermagem, Métodos de Alimentação.

## Abstract

**Objetivo:**

Descrever o processo de construção e validação de um *bundle* para cateterização gástrica em recém-nascidos.

**Métodos:**

Estudo metodológico, desenvolvido entre os meses de abril a outubro de 2023, em três etapas: revisão da literatura, construção e validação de conteúdo por 23 *experts*. O *bundle* para validação, composto por seis itens apresentado em formato *Likert*, foi avaliado on-line pelos *experts*. O Índice de Validade de Conteúdo com valores acima de 0,80 foram considerados para análise.

**Resultados:**

A versão final *do bundle* foi composta por quatro itens. Os cuidados propostos estão relacionados à mensuração do comprimento para inserção do cateter gástrico e métodos para verificação do seu adequado posicionamento em recém-nascidos. A avaliação geral do *bundle* apresentou uma nota global de 10,0.

**Conclusão:**

A validação e implementação do *bundle* poderá contribuir para a qualidade da assistência, visando à prática baseada em evidências e redução de eventos adversos, classificando a sua importância e aplicabilidade para o procedimento.

## INTRODUÇÃO

É notável que a neonatologia possui especificidades e peculiaridades que exigem atendimento especializado. Nas últimas décadas, os avanços técnico-científicos e o aprimoramento nos processos de trabalho têm contribuído significativamente para o aumento das taxas de sobrevida de recém-nascidos (RN) internados em unidades de terapia intensiva neonatais (UTIN) ^(1,2)^.

Nas UTIN, é comum a ocorrência de internações prolongadas, durante as quais os neonatos frequentemente são submetidos a uma série de intervenções realizadas por diferentes profissionais, além de manipulações excessivas e procedimentos invasivos. Esse cenário pode interferir na segurança desses pacientes, tornando-os mais suscetíveis a eventos adversos ^(3)^.

A cateterização gástrica é um procedimento comum realizado no ambiente hospitalar, especialmente nas UTIN. Este procedimento, de competência exclusiva do enfermeiro, não está isento de riscos e implica uma série de decisões que podem comprometer a segurança do neonato durante sua internação ^(4,5)^.

Os cateteres gástricos (CG) são utilizados para várias finalidades, incluindo descompressão gástrica, administração de medicamentos, lavagem gástrica, repouso gástrico após cirurgias, monitoramento de sangramento, bem como para alimentação. Suas principais indicações para neonatos estão voltadas para RN com imaturidade e/ou incoordenação de sucção-deglutição-respiração, assim como para aqueles que apresentam taquipneia ou dispneia. Essas recomendações visam minimizar o risco de broncoaspiração, assegurando uma abordagem segura e eficaz na utilização de CG em neonatos ^(6)^.

O posicionamento inadequado do CG constitui a principal causa de complicações relacionadas ao procedimento de cateterização, conforme evidenciado por estudos em neonatos e crianças, que indicam uma prevalência significativa de eventos adversos, variando entre 47,5% e 59%. Esse posicionamento incorreto pode levar a complicações respiratórias e déficits nutricionais durante a hospitalização. Adicionalmente, os erros de trajetória durante a inserção do cateter, incluindo desvio para a árvore traqueobrônquica e perfuração de tecidos, representam uma categoria crítica de eventos adversos, com potencial para gerar complicações graves e, em alguns casos, fatais para o neonato ^(3,7)^.

A elaboração e implementação de protocolos institucionais padronizados, com base nas evidências científicas mais recentes e centradas no paciente, desempenham um papel importante na obtenção de um cuidado adequado e livre de danos, permitindo a gestão eficaz de processos, tarefas e atividades ^(8)^.

A garantia de cuidado seguro para neonatos em UTIN exige protocolos e estratégias baseadas em evidências que otimizem processos e resultados. Nesse cenário, os *bundles* de cuidados - intervenções padronizadas com base nas melhores evidências - surgem como essenciais para promover segurança e eficácia na assistência. Quando aplicados de forma coordenada, esses *bundles* reduzem eventos adversos e melhoram os desfechos clínicos, especialmente na prevenção de incidentes relacionados ao posicionamento adequado do CG ^(9,10)^.

## OBJETIVO

Descrever o processo de construção e validação de um *bundle* para cateterização gástrica em recém-nascidos.

## MÉTODOS

### Aspectos éticos

Para atender às exigências da Resolução 466/2012 do Conselho Nacional de Saúde, que regulamenta as normas para a realização de pesquisas envolvendo seres humanos, todos os participantes assinaram o Termo de Consentimento Livre e Esclarecido. O estudo foi aprovado pelo Comitê de Ética em Pesquisa, seguindo os critérios da Resolução nº 466/12.

### Tipo e período do estudo

Estudo metodológico, desenvolvido em três etapas: levantamento da produção científica sobre a temática, construção do *bundle* com os cuidados relacionados à cateterização gástrica em RN, incluindo mensuração e verificação do CG, e validação do conteúdo realizada por experts ^(11)^. O estudo seguiu as recomendações do *Standards for Quality Improvement Reporting Excellence* ^(12)^. O desenvolvimento do estudo ocorreu no período de outubro de 2022 a janeiro de 2024, em uma universidade pública do estado de Minas Gerais.

### População, critérios de inclusão e exclusão

O recrutamento dos experts foi iniciado pela Plataforma Lattes, identificando 254 enfermeiros com experiência em neonatologia e/ou pediatria. Após a análise dos currículos, conforme os critérios estabelecidos, foram identificados 28 enfermeiros que atendiam aos critérios de elegibilidade.

Para compor o grupo de avaliadores, os currículos identificados foram analisados segundo critérios específicos para o cálculo do escore: (quatro pontos) para no mínimo quatro anos de experiência clínica na área do estudo (obrigatório); (um ponto) para pelo menos um ano de experiência em ensino clínico na área do estudo; (um ponto) para artigos publicados; (um ponto) para participação de no mínimo dois anos em grupos de pesquisa na área do estudo; (dois pontos) para doutorado na área do estudo; (um ponto) para mestrado na área do estudo e (um ponto) para residência na área do estudo. Para cada ano de experiência clínica ou de ensino, foi adicionado um ponto. Os experts foram categorizados como especialistas júnior, com pontuação mínima de cinco pontos; especialistas master, com pontuação de seis a 20 pontos; e especialistas sênior, com pontuação superior a 20 pontos ^(13)^.

O contato com os experts foi realizado por e-mail, enviando um documento que detalhava os objetivos do estudo e as atividades solicitadas, acompanhado do Termo de Consentimento Livre e Esclarecido (TCLE) e do link para o formulário do Google. Após o consentimento para participação, o acesso ao instrumento era liberado; em caso de recusa, o processo era encerrado. Foram excluídos três experts que não devolveram o instrumento no prazo de 15 dias e dois que o preencheram de forma incompleta. Dos 28 enfermeiros contatados via currículo Lattes, 23 participaram, configurando uma amostra não probabilística de conveniência ^(14)^, com a validação conduzida em uma única rodada.

### Protocolo do estudo

Adotou-se um referencial teórico-metodológico que segue três etapas de procedimentos: teóricos, empíricos e analíticos ^(11)^. Na etapa de procedimento teórico, foi realizado o levantamento das produções científicas referentes à temática. Já o procedimento empírico envolveu a construção do *bundle* para validação de conteúdo por *experts*, e, por fim, a etapa do procedimento analítico consistiu na análise dos resultados de validação.

Para a elaboração dos itens do instrumento, foi realizada uma busca avançada e sensibilizada por estudos nas bases de dados: Cochrane, Literatura Latino-Americana e do Caribe em Ciências da Saúde (LILACS) por meio da Biblioteca Virtual em Saúde (BVS), *Medical Literature Analysis and Retrieval System Online* (MEDLINE) por meio do motor de busca *US National Library of Medicine National Institutes of Health* (PubMed*), Cumulative Index to Nursing and Allied Health Literature* (CINAHL), *Excerpta Medica dataBase* (EMBASE) e Scopus. Foram incluídos estudos primários publicados em português, inglês e espanhol, no período de janeiro de 2013 a junho de 2023, visando identificar estudos mais recentes sobre o tema. Foram excluídos estudos de revisões, reflexões, comentários, resumos de anais, teses, dissertações, artigos de opinião, trabalhos de conclusão de curso, cartas do editor, relatórios, documentos oficiais de programas nacionais e internacionais, capítulos de livros e *e-books*.

A pergunta de revisão foi: “Quais são as evidências disponíveis na literatura acerca das técnicas de mensuração do CG e quais são os métodos alternativos ao exame radiológico para verificar o seu correto posicionamento em RN internados em UTIN?”

As buscas foram realizadas por meio de descritores controlados do *Medical Subject Headings* (MeSH): *“Infant, Newborn”, “Intubation, Gastrointestinal*” e “*Intensive Care, Neonatal*” e dos Descritores em Ciências da Saúde (DeCS): “Recém-nascido”, “Intubação gastrointestinal” e “Terapia Intensiva Neonatal”, combinados pelos operadores booleanos “OR” e “AND”, validados por uma bibliotecária. O levantamento foi realizado de abril a junho de 2023, e os resultados obtidos foram revisados por dois pesquisadores de forma independente. Em situações de discordância, a opinião de um terceiro pesquisador era solicitada, e as decisões eram tomadas por consenso.

A partir das evidências, o instrumento para validação foi desenvolvido em formato *HyperText Markup Language* (HTML) no Google Formulários®, com prazo de 30 dias para resposta, destinado ao preenchimento via web em duas partes. A primeira parte abordou informações para a caracterização e classificação dos especialistas, conforme os critérios ^(13)^: idade, sexo, tempo de experiência profissional, experiência clínica, tempo de docência em enfermagem, aprimoramentos e especializações no âmbito neonatal e publicações na área.

A segunda parte constituiu os critérios de análise dos itens que compuseram o *bundle*, com base em afirmações específicas ^(11)^, incluindo: (1) o instrumento é aplicável e possui instruções claras e exequíveis; (2) as recomendações permitem alcançar o objetivo desejado; (3) os itens expressam uma única ideia e permitem compreensão adequada; (4) o conteúdo é explicitado de forma clara e inequívoca; (5) o instrumento é relevante e atende à finalidade proposta; (6) cada item do instrumento é distinto dos demais e não se confundem; (7) a linguagem é adequada e permite interatividade do conteúdo; (8) o vocabulário é adequado, sem gerar ambiguidades; (9) o vocabulário é condizente com a temática, com conceitos adequados; (10) a formulação do instrumento contribui para uma atitude favorável de utilização e compreensão do conteúdo; (11) o conteúdo é atual, consistente e com profundidade suficiente para a compreensão do tema; (12) a sequência proposta é equilibrada e coerente. A avaliação foi realizada com base em uma escala de *Likert* com opções: concordo fortemente (4), concordo (3), não sei (0), discordo (2) e discordo fortemente (1). Ao final de cada item, havia um campo em branco para que os especialistas opinassem sobre a utilidade do *bundle* na prática e apresentassem “comentários ou sugestões” quanto à concordância semântica para retirada, acréscimo e/ou modificações em cada item ^(11)^.

### Análise dos resultados e estatística

Os dados foram exportados do *Google Formulários*® para um banco de dados no formato *Excel*®, passando por um processo de dupla digitação por dois pesquisadores distintos, para garantir a fidedignidade do registro. O Índice de Validade de Conteúdo (IVC) foi utilizado para avaliar o consenso dos especialistas quanto à representatividade dos cuidados a serem incluídos no instrumento, mediante a soma dos itens “concordo fortemente” ou “concordo”, dividida pelo número total de respostas. O nível de consenso estipulado entre os experts foi de 80%, como recomenda a literatura ^(14)^.

## RESULTADOS

Participaram do estudo 23 especialistas, todos da área de neonatologia e/ou pediatria (100%). A maioria era do sexo feminino (91,3%), com idade média de 38 anos (mínimo de 29 e máximo de 57 anos). O tempo médio de experiência foi de 12 anos. De acordo com a experiência profissional, 21 especialistas (91,3%) foram considerados master, e dois (8,7%) como especialistas seniores.

Em relação à titulação acadêmica (com mais de uma resposta permitida por participante), observou-se que dois possuíam graduação (8,7%), 17 possuíam especialização (74%), seis possuíam residência (26%), cinco tinham título de mestre (21,7%), quatro possuíam título de doutor (17,4%) e um tinha pós-doutorado (4,3%).

O instrumento inicial construído para validação continha seis itens sobre a cateterização gástrica em RN, visando à redução de problemas relacionados à mensuração e verificação do posicionamento adequado do CG, conforme o Quadro 1.

**Quadro 1:** Componentes do *bundle* para redução de problemas relacionados à mensuração e posicionamento do cateter gástrico em RN (versão para validação). Uberaba, Minas Gerais, Brasil, 2023

Componentes
Verificar se o RN tem indicação para inserção do CG
Realizar higienização das mãos antes e após o procedimento
Utilizar a técnica NEMU para mensuração do cateter: Se inserção oral: comissura labial, lobo inferior da orelha ao ponto médio entre o apêndice xifoide e a cicatriz umbilical. Se inserção nasal: ponta do nariz ao lóbulo da orelha ao ponto médio entre o apêndice xifoide e cicatriz umbilical.
Verificar o posicionamento do cateter, aspirar o conteúdo gástrico para examinar a cor e fazer o teste de pH.
Realizar raio-x para confirmação do posicionamento do CG, caso necessário.
Proceder com a anotação de enfermagem no prontuário do procedimento realizado e ocorrências.

*CG: cateter gástrico*

A concordância interavaliadores foi evidenciada quanto aos itens que compuseram o *bundle* proposto e aos 12 critérios de avaliação de conteúdo^(11)^, apresentados na Tabela 1.

**Tabela 1:** Distribuição das respostas dos *experts*, Índices de Validade de Conteúdo por critério e Índice de Validade de Conteúdo total do *bundle* para redução de problemas relacionados à mensuração e posicionamento do cateter gástrico em RN. Uberaba, Minas Gerais, 2023

Itens do *bundle* Critérios gerais de avaliação	Opões de resposta escala *Likert*					Respostas 3 e 4	IVC^*^
	0 n(%)	1 n(%)	2 n(%)	3 n(%)	4 n(%)	n(%)	(%)
Claro e executável			1(4,3)	10(43,5)	12(52,2)	22(95,7)	0,96
Alcança o objetivo				10(43,5)	13(56,5)	23(100)	1,00
Compreensível	1(4,3)		1(4,3)	9(39,1)	12(52,2)	21(91,3)	0,91
Conteúdo Claro	1(4,3)			6(26,1)	16(69,5)	22(95,6)	0,96
Relevante	1(4,3)	1(4,3)		7(30,4)	14(60,9)	21(91,3)	0,91
Os itens são distintos				10(43,5)	13(56,5)	23(100)	1,00
Conteúdo claro ao público alvo		1(4,3)	1(4,3)	8(34,8)	13(56,5)	21(91,3)	0,91
Linguagem clara ao público alvo				8(34,8)	15(65,2)	23(100)	1,00
Linguagem adequada ao conteúdo			2(8,7)	8(34,8)	13(56,5)	21(91,3)	0,91
Contribui para a compreensão do conteúdo	1(4,3)		1(4,3)	9(39,1)	12(52,2)	21(91,3)	0,91
Conteúdo atual	1(4,3)		1(4,3)	8(34,8)	13(56,5)	21(91,3)	0,91
Sequência coerente			2(8,7)	07(30,4)	14(60,9)	21(91,3)	0,91
IVC Total†							0,94

* *IVC: Índice de Validade de Conteúdo por item; IVC total†:Índice de Validade de Conteúdo geral do bundle.*

Quanto à concordância interavaliadores, analisada primeiramente para cada item do *bundle* proposto, todos os critérios avaliados foram classificados como de “quase perfeita concordância” (0,81 a 1,00). A análise do IVC por critério revelou que três itens (25%) receberam a pontuação máxima de 1,00. Dois itens (16,7%) alcançaram a pontuação de 0,96, enquanto sete itens (58,3%) obtiveram a pontuação de 0,91.

Obteve-se o valor de 0,94 (quase perfeita concordância) para o IVC total do *bundle*, o que subsidiou caracterizá-lo como um constructo válido em seu conteúdo. Não foi necessária uma reavaliação pelos especialistas. Assim, o aprimoramento do constructo, após a incorporação das sugestões dos avaliadores, foi conduzido apenas como uma devolutiva.

Após analisar as sugestões dos especialistas, foram implementadas melhorias no *bundle* para torná-lo mais prático e de fácil compreensão. Optou-se por remover os itens um e seis do instrumento inicial, uma vez que a introdução do cateter será realizada apenas em RN com indicação clínica, e a anotação de enfermagem é considerada obrigatória em qualquer procedimento. Dessa forma, a versão final do *bundle* foi consolidada em quatro itens, refletindo uma abordagem mais focada e alinhada com as necessidades específicas do procedimento de cateterização gástrica em RN. Essa simplificação visa otimizar a aplicação prática do *bundle*, mantendo sua eficácia e relevância para a equipe de enfermagem.

A versão final do *bundle* sobre cateterização gástrica em RN para redução de problemas relacionados à mensuração e posicionamento do CG em UTIN está apresentada no Quadro 2, incorporando as alterações sugeridas pelos *experts*.

**Quadro 2:** Versão final do *bundle* para redução de problemas relacionados à mensuração e posicionamento do cateter gástrico em RN. Uberaba, Minas Gerais, Brasil, 2023

*Bundle* para redução de problemas relacionados à mensuração e posicionamento do CG em RN
Realizar higienização das mãos antes e após o procedimento.
Utilizar a técnica NEMU para mensuração do cateter, considerando o comprimento total, incluindo os orifícios: Se inserção oral: comissura labial, lobo inferior da orelha ao ponto médio entre o apêndice xifoide e a cicatriz umbilical. Se inserção nasal: ponta do nariz ao lóbulo da orelha ao ponto médio entre o apêndice xifoide e cicatriz umbilical.
Verificar o posicionamento do cateter: aspirar o conteúdo gástrico para examinar a cor, fazer o teste de pH (valor de referência: ≤5,5) e medir o comprimento do cateter que ficou exposto.
Realizar raio-x em caso de dúvidas do posicionamento do CG.

*CG: cateter gástrico.*

## DISCUSSÃO

A elaboração e aplicação de *bundles* têm se destacado por aprimorar os processos e os resultados dos pacientes, além de prevenir agravos à saúde, especialmente aqueles considerados evitáveis^(15-17)^. A adoção dessa abordagem contribui para uniformizar as ações da equipe de enfermagem, reduzindo as disparidades na prestação dos cuidados.

A validação do processo confere robustez metodológica ao *bundle*, sendo crucial avaliar a perspectiva da população-alvo para aprimorar a compreensão dos seus elementos ^(11,18)^. Torna-se evidente a necessidade de ferramentas simples e diretas na prática clínica, levando em conta as demandas e os recursos disponíveis nos serviços de saúde.

Desse modo, um dos cuidados elencados no *bundle* foi a “higiene das mãos”, frequentemente listado como o primeiro item na maioria dos protocolos de segurança em saúde. Esse cuidado é reconhecido como uma medida de baixo custo, simples e altamente eficaz para evitar infecções. No entanto, é importante considerar as limitações na adesão por parte dos profissionais ^(19)^.

Deve-se reconhecer que há uma discrepância anatômica na distância entre a ponta do nariz e o lóbulo da orelha para a inserção do cateter via nasal, em comparação com a distância da comissura labial até o lóbulo da orelha para a inserção via oral. Embora essa diferença seja mínima para o RN, pode impactar o correto posicionamento do cateter. Portanto, é de suma importância conduzir mais pesquisas que validem as medidas anatômicas externas de referência para a inserção do CG. Essa abordagem visa prevenir complicações decorrentes do posicionamento inadequado e aprimorar as práticas clínicas relacionadas a esse procedimento ^(20)^.

Os critérios para escolha da via de inserção do CG devem ser cuidadosamente determinados, levando em consideração o desenvolvimento da coordenação neuromuscular de cada RN. Por exemplo, o cateter oral gástrico pode ser utilizado nos primeiros dias, sendo posteriormente substituído pelo cateter nasal gástrico após a estabilização respiratória do bebê. Essa abordagem personalizada considera as necessidades específicas de cada RN, visando garantir um procedimento seguro e eficaz ^(8)^.

Considerando a necessidade de assegurar a posição gástrica adequada do cateter e prevenir complicações, as diretrizes do Programa de Reanimação Neonatal da Academia Americana de Pediatria, da Associação Nacional de Enfermeiros Neonatais Americanos e do Ministério da Saúde, por meio do Manual de Boas Práticas e do Manual de Reanimação Neonatal da Sociedade Brasileira de Pediatria, atualmente recomendam a adoção do método NEMU ^(21-23)^.

Os ajustes sugeridos na validação do instrumento permearam três itens do *bundle* e estiveram relacionados a melhorias na compreensão dos cuidados.

A primeira sugestão abordou a consideração dos orifícios do cateter. A ausência de orientações sobre as precauções necessárias em relação a cateteres com múltiplos orifícios na extremidade distal, nos estudos revisados, ressaltou uma lacuna significativa. Os estudos mencionam o “comprimento de inserção”, sem esclarecer se este deve incluir ou excluir os orifícios, detalhe crucial que deveria ser explicitado. Diante dessa falta de informação, a recomendação é considerar o comprimento total, incluindo os orifícios, ao realizar a medição a partir da extremidade distal. É importante enfatizar a utilização de um cateter especialmente desenvolvido para essa finalidade, como o CG infantil, para assegurar a precisão da medida. O uso de cateteres uretrais ou de aspiração traqueal, por exemplo, pode comprometer a precisão devido às variações na padronização dos orifícios ^(6)^.

A segunda sugestão refere-se aos métodos de verificação para confirmar o posicionamento do CG. Quanto à precisão e segurança dos métodos alternativos ao exame radiológico, a ausculta e a aspiração isoladas não são recomendadas. A ausculta da região epigástrica é considerada pouco confiável, sendo desaconselhada devido à dificuldade em diferenciar entre o som de ar instilado no estômago e no pulmão. No caso da aspiração de secreção e avaliação de sua cor e aparência, essas medidas podem ser sensíveis, mas imprecisas, para confirmação do posicionamento do cateter. Isso se deve à falta de especificidade estabelecida, uma vez que secreções endotraqueais e brônquicas podem apresentar coloração e aspecto semelhantes aos das secreções gástricas. Recomenda-se a combinação de dois ou mais métodos de verificação para aumentar a confiabilidade do processo^(24,25)^.

Atualmente, há consenso entre os especialistas de que a verificação do pH com tiras reagentes à beira do leito é o método mais seguro e recomendado como primeira opção para confirmar o posicionamento do CG. A interpretação dos resultados sugere que valores de pH ≤5,5 indicam um posicionamento gástrico correto, enquanto valores >5,5 requerem confirmação radiológica ^(24)^.

É importante destacar que a disponibilidade de tiras de pH pode não ser uma realidade em alguns hospitais brasileiros. Assim, a escolha do método para avaliar o trajeto e a posição do CG dependerá dos recursos disponíveis e do planejamento dos cuidados de enfermagem em cada instituição ^(6)^. Nesse contexto, é crucial considerar a implementação desse método na prática clínica hospitalar, tendo em vista sua recomendação e o baixo custo das tiras de pH.

A terceira sugestão abordou a realização de raio X para confirmar o posicionamento do CG, estabelecendo diretrizes para a conduta diante da identificação de problemas. Em casos de incerteza sobre a posição correta do CG, a recomendação é que a substituição, reavaliação e/ou confirmação radiográfica sejam consideradas antes da administração de qualquer conteúdo pelo cateter ^(8,24)^.

### Contribuição para área da enfermagem e saúde

Os achados na literatura revelam divergências significativas na prática de mensuração do CG e nos métodos alternativos para verificar o posicionamento, o que é preocupante. Tais resultados destacam a urgência de incorporar estratégias de medição e verificação da posição do cateter nos protocolos institucionais, além de enfatizar a importância da educação continuada dos profissionais. Essas iniciativas são cruciais para viabilizar mudanças nas práticas individuais. A expectativa é que este estudo provoque reflexões e discussões sobre a prática de cateterização gástrica nas Unidades Neonatais, subsidiando as ações da enfermagem e os cuidados na área da neonatologia.

### Limitação do estudo

Atribuiu-se, como limitação deste estudo, a escassez de evidências sobre a temática provenientes de ensaios clínicos randomizados, que são considerados padrão ouro na avaliação da eficácia e segurança de intervenções em saúde.

## CONCLUSÃO

Este estudo possibilitou a construção e validação de conteúdo do *bundle*, por meio da avaliação de 23 experts, destinado ao procedimento de cateterização gástrica em RN. Os cuidados abordaram aspectos relacionados à mensuração do comprimento de inserção do CG e ao seu correto posicionamento. Todos os itens do *bundle* apresentaram IVC superior ao recomendado, e pequenos ajustes foram implementados para aprimorar a compreensão geral. Os participantes reconheceram a relevância do instrumento para a prática clínica. A adoção desse *bundle* poderá contribuir para a qualidade da assistência e das práticas de enfermagem no atendimento ao RN, surgindo como ferramenta complementar aos cuidados prestados.
